# “I feel like it gets worse as I get older”: perspectives of peri-postmenopausal women with PCOS

**DOI:** 10.3389/fgwh.2025.1588505

**Published:** 2025-08-12

**Authors:** Pamela J. Wright, Cynthia F. Corbett, Robin M. Dawson, Charlotte Burts

**Affiliations:** Department of Biobehavioral Health & Nursing Science, Advancing Chronic Care Outcome Through Research and iNnovation (ACORN) Center, College of Nursing, University of South Carolina, Columbia, SC, United States

**Keywords:** polycystic ovary syndrome, menopause, perimenopause, postmenopausal, women's health, qualitative research

## Abstract

**Introduction:**

Polycystic ovary syndrome (PCOS) is a chronic endocrinopathy that transcends the reproductive years. Peri-postmenopausal women with PCOS remain at cardiometabolic risk or subsist with established comorbidity while continuing to contend with persistent PCOS signs and symptoms such as hirsutism. Evidence based information for peri-postmenopausal women with PCOS is scant. The purpose of this qualitative study was to explore the physical, psychosocial, and healthcare perspectives of peri-postmenopausal women with PCOS.

**Methods:**

Peri-postmenopausal women with PCOS aged ≥43 years (*n* = 29) were recruited using ResearchMatch to participate in a virtual interview. Interviews were conducted using a semi-structured guide. The interviews were transcribed, de-identified, and analyzed using the steps of reflexive thematic analysis.

**Results:**

Participants were aged 52.5 (±6.6) years, mostly White (69.0%), and highly educated with 62.0% having at least a college degree. In terms of health, most participants were perimenopausal (*n* = 20; 69.0%), categorized as “obese” (BMI ≥30) (*n* = 20, 69%), had ≥3.0 comorbidities (*n* = 26; 90%), and took ≥5 prescribed medications (*n* = 17; 58.0%). Five overall themes were identified: (1) Déjà vu?, (2) Sociocultural Stigma, (3) Trauma Experiences, (4) Self-Advocacy, and (5) Resilience vs. Resignation.

**Discussion:**

The identified themes highlight the need for a more proactive, trauma-informed, culturally tailored healthcare approach that provides education and support in managing PCOS over the lifespan. Women's needs include resources after adverse pregnancy outcomes and routine screening of emotional health, with subsequent treatment when indicated. The findings indicate a need for healthcare provider communication and sensitivity training. Women with PCOS could benefit from advocacy and resilience training. As a relatively unexplored area of women's health, more research is needed to address the needs of older women with PCOS and develop and test programs that train healthcare providers to deliver patient-centered care and equip women with the information and resources to properly manage PCOS and cop effectively over their life course.

## Introduction

Polycystic ovary syndrome (PCOS) is a chronic, complex, heterogenous endocrinological condition that affects 15%–20% of the population across all races and ethnicities globally (1). According to Rotterdam criteria, the accepted criteria by the international PCOS guidelines, PCOS is diagnosed when at least two of these three conditions are present: (1) clinical or biochemical hyperandrogenism, (2) irregular menstrual cycle, and (3) polymorphic ovarian morphology (2). Among women with PCOS, ∼50% have obesity (3), ∼60% have visceral adiposity (3), 70% have dyslipidemia (4), and ∼80% have insulin resistance (3), all which increase their risk of cardiometabolic diseases (3) and reproductive cancers (5) by up to 50% compared to that of the general female population. Clinical features plus symptoms such as hirsutism (excessive male-pattern hair growth) and acanthosis nigricans (dark, thick, velvety skin in body creases) negatively impact health-related quality-of-life (HRQL) among women with PCOS (6) especially in the psychological domain (7). Women with PCOS are 3–8 times more likely than women without PCOS to have depressive symptoms (8) and 8.5 times more likely to attempt suicide (9).

As a chronic condition, PCOS transcends the reproductive years. Peri-postmenopausal women with PCOS have a higher prevalence of visceral obesity, insulin resistance, and elevated triglycerides as compared to age-matched women without PCOS (10). Additionally, hyperandrogenism, a defining diagnostic criterion of PCOS, persists over the lifespan, promoting the likelihood of cardiometabolic conditions while perpetuating undesirable signs such as hirsutism and prompting new signs such as alopecia (male-pattern balding) (11). Androgen levels may also increase as women enter menopause, while at the same time, estrogen levels decrease (12). Thus, peri-postmenopausal women remain at cardiometabolic risk and/or subsist with established comorbidity while continuing to manage persistent PCOS signs and symptoms. Additionally, the menopausal transition is on average four years longer than age-matched controls without PCOS (11), indicating an extended experience with menopausal symptoms such as irritability and vaginal dryness. Currently, most literature about PCOS considers the reproductive years. There is little evidence-based information about the PCOS experience during the peri-postmenopausal years, especially the perspectives of women with PCOS. In our previous study involving peri-postmenopausal women with PCOS, we found a need for more in-depth interviews to assess their psychosocial and emotional needs (13). Thus, the purpose of this qualitative study was to explore the physical, psychosocial, and healthcare perspectives of women with PCOS during and after the menopausal transition.

## Materials and methods

### Study design

An interpretive qualitative study was conducted using a semi-structured guide to interview peri-postmenopausal women with PCOS (*n* = 29).

### Recruitment

Participants (*n* = 29) were recruited using ResearchMatch, a free and secure web-based National Institutes of Health (NIH) clinical research registry allowing access to participants across the United States (US). Inclusion criteria were peri-postmenopausal women aged 43 years or older with PCOS whose primary language was English. Since the reproductive age is defined as18–43 years in most PCOS research articles (14, 15), 43 years was selected as the minimum age for recruitment. Perimenopause was defined as deviation from one's typical menstrual cycle pattern (16). Since women with PCOS tend to have irregular menstrual cycles, especially without oral contraception, deviation was defined as a change in their personal expected cycle schedule and characteristics. Menopause was defined as one year without a menstrual cycle (16). An exclusion criterion was surgically induced menopause, that is, the removal of both ovaries before the onset of natural menopause (17).

### Research ethics and consent

The study was conducted in accordance with the principles stated in the Declaration of Helsinki. In accordance with 45 CFR 46.104(d)(2) and 45 CFR 46.111(a)(7), the University of South Carolina Institutional Review Board (IRB) provided an “exempt” status (Pro000124188; 10/3/2022) for the study. Exempt status was granted because the study involved interviews, and data were recorded such that participants could not be identified. According to federal guidelines, the IRB waived the requirement for written informed consent, as this document would have been the only link to the participant. Additionally, the research posed no harm to the participants. Verbal informed consent was obtained before each interview after explaining the purpose, procedures, and risks/benefits of the study to the participant.

### Measures

#### Demographics

The demographic questionnaire included age, height, weight, age or year at PCOS diagnosis, last menstrual period (LMP), race/ethnicity, educational attainment, employment, marital status, number of children, comorbidities, and current medication use.

#### Interview

The interview was conducted using a semi-structured interview guide (Supplementary Appendix A) created by three researchers (PJW, CFC, RMD) through the lens of the bioecological conceptual model. The bioecological model posits that human development occurs through progressively more complex reciprocal interactions with one's biopsychosocial characteristics, the environment, and social contexts over time (18).

### Data collection and management

Once potential participants agreed to be contacted, the primary researcher emailed them. The email included a demographic questionnaire with screening questions (e.g., last menstrual period). Potential participants who returned the questionnaire were again contacted via email to schedule an interview with the primary researcher using either the TEAMS or Zoom platform. If there was any question about eligibility, the primary researcher and the potential participant discussed the woman's menstrual and medical history to confirm that the study inclusion and exclusion criteria were met. Following receipt of informed verbal consent and prior to starting an interview, the primary researcher (PJW), a doctoral prepared female nurse scientist with several years of experience recruiting and working with women with PCOS, introduced herself and stated her credentials, vocation, and experience. Interviews were not video recorded. However, the transcription feature was enabled to capture participants' answers. Transcripts were downloaded, de-identified, and stored in a secure shared file (Microsoft OneDrive). Files were kept secure during transfer with SSL/TLS encryption protocols offered by the University's computer network. There was no follow-up with participants.

### Data analysis

Data were analyzed using the steps of reflexive thematic analysis: data familiarization, generation of initial codes, grouping of codes into broader patterns, data comparison for consistency and accuracy, identification and naming of themes, and manuscript production (19). The research team collectively reviewed, discussed, and identified codes in the first three transcripts to initiate a coding manual. Then, the researchers were randomly assigned the remaining transcripts, such that each transcript was reviewed and coded by at least two researchers. The first author (PJW) reviewed all transcripts. The research team then collectively discussed codes, grouped them into categories, and then into broader themes. These themes were discussed and refined via data immersion, reflexive thought, and group discussion.

### Rigor

Rigor was strengthened through the reflexivity of the four researchers and the continuous reassessment and reiteration of coding. An audit trail was maintained via documentation and regular communication among the four researchers. COREQ (Consolidated Criteria for Reporting Qualitative Research) guidelines were followed to also increase rigor and credibility and enable explicit and comprehensive reporting of the findings (Supplementary Appendix B) (20). The de-identified data supporting the findings of this study are available from the primary author by reasonable request.

## Results

**Figure 1:**
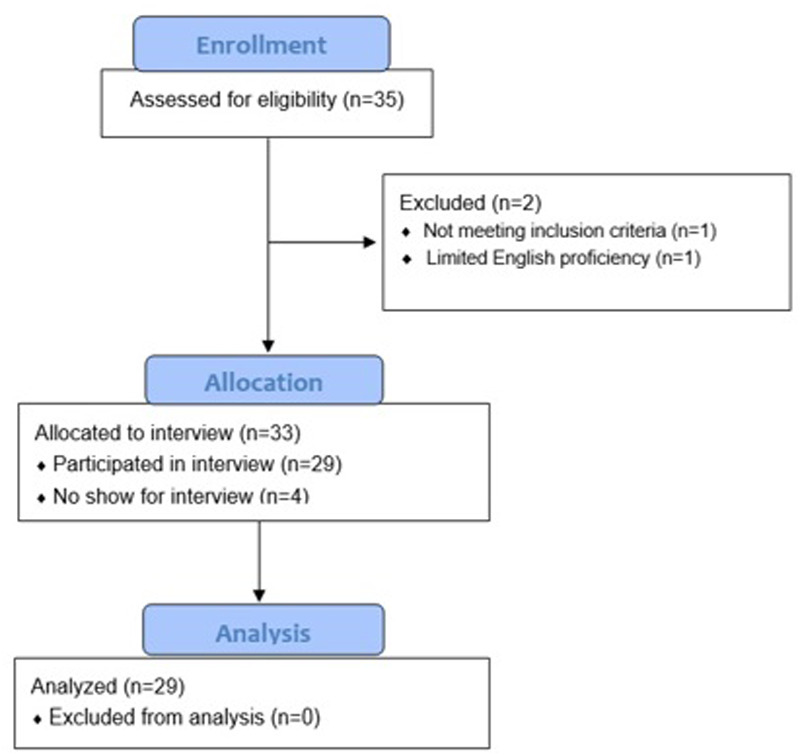
Consort flow diagram.

Recruitment efforts led to 35 potential participants, two of whom did not meet inclusion criteria (e.g., limited English proficiency). The remaining potential participants (*n* = 33) were scheduled for virtual interviews; however, four did not attend the virtual meeting and did not respond to follow-up invitations to reschedule. See Figure 1.

**Figure 2:**
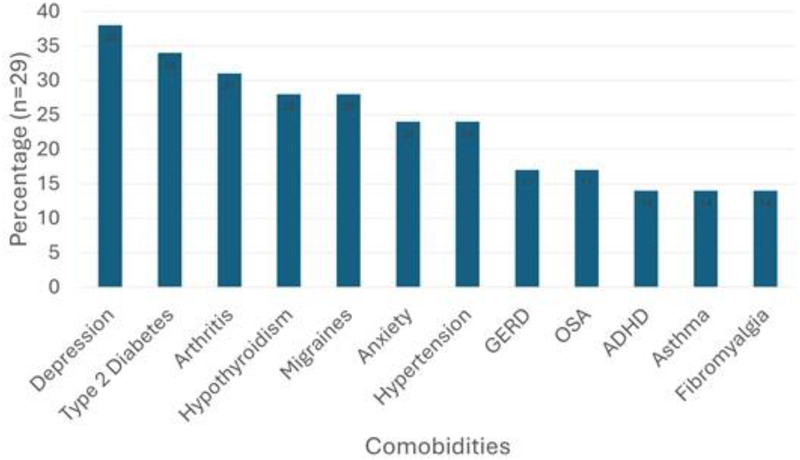
Common comorbidities among sample (*n* = 29).

Participants were aged 52.5 (±6.6) years, mostly White (69.0%), and educated with 62.0% having at least a college degree (Table 1). In terms of health, most participants were perimenopausal (*n* = 20; 69.0%), categorized as “obese” (BMI ≥30) (*n* = 20, 69%), took ≥5 prescribed medications (*n* = 17; 58.0%), and had ≥3.0 comorbidities (*n* = 26; 90%) (Figure 2). All participants were receiving medical care for PCOS and/or associated comorbidities, with three (10%) attempting to find a new healthcare provider.

**Table 1:** Demographic and health characteristics of peri-postmenopausal women with PCOS (*n* = 29).

Characteristics	Mean (±SD)
Age	52.5 years (±6.6)
Weight	214.0 lb (±64.6)
BMI	35.1 kg/m^2^ (±10.7)
Years since Diagnosis	22.5 (±5.1)
Characteristics	*n* (%)
Race/Ethnicity	
• African American/Black	7 (24.0)
• Latino	1 (3.5)
• White	20 (69.0)
• Mix of Two	1 (3.5)
Education	
• High School	5 (17.0)
• Some College	6 (21.0)
• College	9 (31.0)
• Masters	6 (21.0)
• Doctorate	3 (10.0)
Employment	
• Full-time	9 (31.0)
• Part-time	2 (7.0)
• Not working	18 (62.0)
Marital Status	
• Single	5 (17.0)
• Married	13 (45.0)
• Divorced	10 (34.5)
• Widowed	1 (3.5)
Children	
• 0	13 (45.0)
• 1–2	11 (38.0)
• 3–4	5 (17.0)
Comorbidities	
• 0	1 (3.5)
• 1–2	2 (7.0)
• 3–4	11 (38.0)
• 5–8	10 (34.5)
• ≥9	5 (17.0)
Medications	
• 0	2 (7.0)
• 1–2	4 (14.0)
• 3–4	6 (21.0)
• 5–8	8 (27.0)
• ≥9	6 (21.0)
• Prefer not to answer	3 (10.0)

Five overall themes were identified: (1) Déjà vu?, (2) Sociocultural Stigma, (3) Trauma Experiences, (4) Self-Advocacy, and (5) Resilience vs. Resignation. Quotes illuminating the themes are presented as stated by the participants and without any editorial changes.

### Déjà vu?

Déjà vu is the feeling that a situation has been previously experienced when it has not (21) … or has it? Participants compared PCOS symptoms from earlier years to those of the peri-postmenopausal years, stating they seem to be reliving the past. The women reported persistent hirsutism (*n* = 21; 72%), recurrence of acne (*n* = 11; 40%) irregular menstrual cycles (*n* = 17; 59%), increased weight gain (*n* = 20; 69%) and fatigue (*n* = 9; 31%). They also reported an onset of new symptoms such as alopecia (*n* = 4; 14%), and the development of chronic comorbidities (*n* = 26; 90%) such as type 2 diabetes and hypertension. Several participants stated the only real difference in their symptoms and health concerns at their current age as compared to when they were in their 20 s was that they no longer had to worry about fertility.

And, of course my periods are crazy again. Sometimes, they're barely there; sometimes they're horrible. (P22)

I feel like it gets worse as I get older, and it felt pretty manageable at first when I first started having it, but then the weight started getting harder to deal with and trying to get off, and I’d have to put in like twice the effort. (P6)

Now I got most all the symptoms that they list. You know the lifelong complications from PCOS too. (P1)

With the onset of the menopausal transition, participants reported a more unpredictable menstrual cycle length and quantity, low libido, hot flashes, sleep difficulties, and mood changes. When seeking answers and treatments, participants noted limited information on menopause, and especially menopause in the presence of PCOS. Some women were misinformed that having children cured PCOS, and other women were uncertain if they still had PCOS once menopausal. As in the days prior to their PCOS diagnosis, participants were again visiting multiple providers for answers and feeling dismissed.

I've gone through 2 endocrinologists and an OB and my family doctor, and nobody's listening to me. They act like that menopause doesn't even exist. (P8)

Menopause is just as big as the other stages. But it's like once again, it's not being talked about, which is frustrating because obviously, we want, you know, people, females like me, want answers. (P8)

Well, I just, I think that they don’t really view them (PCOS and menopause) as real conditions. I mean, this is from the time of being young till now, like it's just kind of overlooked as not being real conditions, sort of like fibromyalgia, which I also have. It's just all in your head. It'll go away. (P19)

Many participants reported that most healthcare professionals exhibited poor patient-provider communication and insensitivity when addressing their healthcare needs during the menopausal transition.

The thing is, patients are not being heard, regardless of what it is that you have. It seems to be the biggest thing that people that I've talked to is doctors aren't listening. The whole frustrating thing is that lack of communication between doctors and families. (P8)

I've heard from different people, including my doctor, that it's a natural part of life. Well, okay. So? And, just to get through it. That's totally insensitive. You can guarantee a man wouldn't be suffering through it. (P10)

My doctor didn't have the emotional or sympathetic or empathetic portion. I would say that doctors need to go through a mandatory training about how to deal with their patients in a sympathetically, empathetic manner. (P21)

Participants perceived weight blaming and weight shaming from their healthcare providers throughout their PCOS journey. When diagnosed with PCOS, participants reported recurrent advice to “just lose weight”. After having children, the weight loss message was not as frequently heard from providers. However, once women started asking their healthcare providers about the menopausal transition, greater emphasis was again placed on weight loss.

I finally got tired of going to gynecologists who were like it was like I didn't have any discipline, and I wouldn't quit eating. And, they'll just be like you need to lose weight. It's the same thing now. (P7)

I would give a complaint, and they would be like, you're fat. Lost 115 pounds. Came back still with the same problem. They're like because you're still fat. They say I'm still fat. (P22)

Everybody is quick to kind of dismiss you and think, oh, you're just making excuses for your weight. (P8)

Consequently, due to inadequate information and unsatisfying interactions with healthcare professionals, many of the participants pursued healthcare information and social support via the Internet.

It (online sites) was a really great resource for me, because I can turn to other women who have this and see what's worked for them. (P7)

I use social media I try to find more on the postmenopause, and it's very, very limited. (P8)

But when I do talk to my doctor, I say I consulted with Dr. Google. You're my second opinion. (P9)

### Sociocultural stigma

Sociocultural stigma refers to the negative attitudes and beliefs that society holds towards certain groups or behaviors based on characteristics or behaviors that often lead to discrimination and social exclusion within a cultural or social contexts (22). Sociocultural stigma can be internalized creating a negative feeling of being different compared to others in society and contrary to social norms (23). Sociocultural gender norms were discussed by many of the participants, mostly about the expectations of women regarding roles, such as marriage, pregnancy, and parenting. The participants who experienced infertility (*n* = 13; 45%) expressed that PCOS “robbed” them of the look and function of femininity.

My parents were there but their beliefs made me uncomfortable talking to them about my issues. I was embarrassed and afraid because they had already made remarks through the years that my weight and acne would keep me from marrying. (P10)

And, I felt like for a lot of women, it (not being a mother) plays into some of the depression that women report. Yeah, because women are the mothers, and if you can't be a mother, what are you? That's society's view by kind of really high level. (p27)

You're expected to be a nurturer; you're expected to be, you know, the family planner. You're expected to do all the emotional labor in the family a lot of times. You're expected to look nice and be pleasant and go along with whatever is being said, you know, don’t cause problems. I feel like a lot of that is still prevalent in society. I think a lot of women fall prey to that societal expectation with the whole you go to school, you find your mate, you get married, you get a house, you have children. It's like this whole plan that's already made for you. (P26)

Several participants explained that their femininity or sense of being a woman was negatively influenced by PCOS or that they needed to “hide” prior to the menopausal transition, and then aging made them feel completely invisible as a woman.

So, PCOS made me feel like less of a woman and that's when I felt like a kind of an attack on my femininity, and I think that's one of the most challenging things about it. It makes you feel like less of a woman because it gives you things that are really associated with the male entity. Now that I'm older, no one sees me. (P7)

I do hate being invisible to people. I feel in some ways as you get older as a woman you become unseen in some ways, and it's like, so the only time within life you deserve attention is if you're young and attractive, you know. (P7)

Aging is a double standard for women. Women are unseen, invisible, irrelevant. (P16)

As such, many of the participants have struggled with compromised self-image, both in their premenopausal years and now.

So, with my hair, my stomach, aging, and things like that, I do feel self-conscious. (P21)

And, then hiding your hair, you know. You also lose your hair, you lose your edges, and so you can't really wear hair styles. You can't wear wigs all the time. So, just kind of hiding, hiding yourself, basically hiding. You gotta hide and put on a different face so they can’t randomly see the real you. Because PCOS is everywhere. It's in your face. It's in your body. (P6)

### Traumatic experiences

Many of the participants described medical traumatic experiences at the systemic and interpersonal levels that resulted in long-term effects on their physical and mental health. Several stories were relayed about medical gaslighting, which is an act by a healthcare provider that dismisses, diminishes, or discredits a patient's illness experience (24).

When you're dismissed and I would even say, gaslighting, so much that you still know something's wrong. (P18)

I kind of ignored it for a while because I wasn't given any information or even a thought about what I should do, if anything. I feel some regret that maybe I could have had a different life if I had treated those things. I mean, I just didn't have any idea. I thought it was only about I might have trouble with pregnancy, which seems to be what was most important for the doctors to say. They didn't care and they didn't tell me anything else. (P27)

So, I was just trying to figure out what was going on, and it was like I kept pushing. I kept pushing and pushing and pushing because my OB kind of blew me off like it was nothing. (P8)

Another source of medical trauma was institutional betrayal. Institutional betrayal is maltreatment and/or a lack of response or a negative response to a patient's healthcare concern by a healthcare provider (25). Medical experiences perceived as traumatic led to medical distrust, healthcare avoidance, and nonadherence with a prescribed treatment plan.

After a very painful experience: We (her & mother) left there crying, and my mom threw the prescription out the window. It was so traumatic and I never liked a male doctor after that. (P7)

When I had my first female exam, the doctor was inconsiderate and rough. It was horrible. It was humiliating. (P10)

Significant sources of trauma for many participants were infertility, recurrent pregnancy loss, and long and costly rounds of fertility treatment.

I'm still trying to come to terms with the fact that I'll never genetically be a parent. Uh, yeah, it's been very painful and was a time-consuming and costly battle. (P2)

“We tried and tried and tried. I had miscarriage after miscarriage. I reached my emotional point and I was done. The depression keeps going today”. (P25)

I did feel embarrassed and a bit inadequate because I couldn't have children and people would, you know, after they found out about my miscarriage, I would get a lot of sadness and pity, and that would only just make me feel even worse because I didn't want them to be sad about my situation. I was sad enough. I am sad. (P21)

### Self-advocacy

Many participants (*n* = 18, 62%) talked about the need for self-advocacy, especially among younger women today. Most of the women wished they had self-advocated during their younger years but did not due to a lack of knowledge or the patient-provider power imbalance.

At the time, I did not have enough knowledge to advocate for myself. (P21)

“… trying to be stronger in health care”. I think it's scary. When you go in as a patient, you’re really the customer, but that's not the power dynamic. The care provider has all of the power. I would try to empower her (my former self) to be more assertive in her health care and either call professionals out or seek new health care, instead of just accepting either being dismissed or whatever. (P7)

While many of the participants were proponents for self-advocacy among women, several lamented about their current struggles as an older woman to self-advocate in the healthcare environment.

So, I feel like being a self-advocate is needed and empowering but is difficult to do, even now, because they don't listen. (P9)

I wish I had advocated for myself more than I had. I try not to be so intimidated by the medical system and really advocate and push more of I know my body. And this just isn't right. There's something wrong, and it's just not right. And I think also being a woman, especially because when you go to a male doctors, sometimes they think it's just women. (P22)

### Resilience vs. resignation

Resilience is the process of adapting well in the face of adversity, trauma, or significant sources of stress (26). Almost half of the participants (*n* = 13; 45%) discussed the need to develop coping skills or mechanisms to build resilience. Coping strategies mentioned included a sense of humor, counseling, cognitive reframing, positive self-talk, mindfulness, exercise, and social interaction.

It's bothering me, but actually, for me, aging makes you happy that you've had all the experiences that you've learned, and hopefully that you know you're more able to cope. (P4)

I cope way better than I did in the beginning because I have meds and I have more confidence in myself. I care a lot less about others' opinions about me or my life. (P10)

I feel like I survived because of different tools, such as dialectical behavior therapy and medication. So, like a combination of things like exercise and living healthier. (P1)

Resignation is the passive acceptance of undesirable but inevitable conditions, often accompanied by a sense of helplessness (27). Nine (31%) participants expressed resignation stating they have no other choice and reporting continuous battles with depression.

It's almost this pushing back on acknowledging that I have it, or that I have to still keep dealing with it. That is still affecting me. I cannot escape it. (P15)

Learning, kind of learning how to deal with things that won't change. Then you get older. (P18)

I've been struggling with depression and stuff for the last, oh, gosh, most of my life since, probably about 20 years old. You have to just accept that it will never be better. (P20)

## Discussion

The menopausal transition is a transformative life phase for women with and without PCOS. Women experience shifts in responsibilities and priorities as adult children leave the home, elderly parents require care, and job demands increase or cease with retirement. Women with PCOS spoke to these changes in context of the PCOS experience, which included persistent PCOS symptoms such as hirsutism and the onset of chronic comorbidities associated with PCOS (e.g., type 2 diabetes and autoimmune disorders). PCOS is a chronic health condition that affects women during and after the menopausal transition. The results of this study provided information about the peri-postmenopausal journey for women with PCOS, including their physical, psychosocial, and healthcare perspectives.

### Déjà vu?

When women with PCOS started having perimenopausal symptoms and seeking care from their healthcare professionals, they realized the experience was similar to the time during and shortly after their diagnosis with PCOS. After the early reproductive years of 18–35 years, ovulatory function improves in many women with PCOS who report a predictable menstrual pattern, especially if taking oral contraceptives (28). However, perimenopause changes this norm. As during their PCOS diagnostic experiences, the women in this study consulted with several healthcare providers and received little if any information about their symptoms or the menopausal transition. This finding is consistent with the results of a qualitative study among adult women with PCOS in Canada, in which perimenopausal women also reported seeing multiple healthcare providers and stated a lack of information and guidance, creating a barrier to symptom management and prompting them to either seek information elsewhere or self-treat (29). These sentiments were echoed by a sample of postmenopausal women without PCOS in a cross-sectional, mixed methods study. Women indicated that healthcare providers had a lack of knowledge or empathy for the menopausal transition, which left the women feeling uninformed and unsupported as they enter the menopausal transition (30).

A few participants described exceptional care due to the healthcare provider's ability and willingness to actively listen, empathize, and provide resources. However, many participants in this study agreed that patient-provider communication is generally poor, with healthcare providers often perceived as insensitive, lacking empathy, and weight blaming. Weight blaming felt harsher for women in the peri-postmenopausal years as many of the women had higher bodyweights compared to their younger years. Several other researchers have reported weight blaming among women with PCOS across all ages (29, 31, 32). In our study, many participants were prescribed weight loss but not provided with guidance or resources to accomplish this goal. Other evidence has shown that obesity rates in women with PCOS are 4-fold higher in women with PCOS than those without PCOS, with excess weight beginning as early as age five and increasing throughout the lifespan (33). Additionally, women with PCOS are arguably at higher risk for cancer and cardiometabolic sequalae that include dyslipidemia, hypertension, and type 2 diabetes (34, 35), although there is a need for more longitudinal studies that examine cardiometabolic outcomes across the lifespan (33). These data highlight the need for additional education and training in PCOS and the management of PCOS among healthcare professionals. Findings from a qualitative study involving focus groups and interviews of women with PCOS revealed that both interactive (engagement with healthcare providers) and functional health literacy (skills to enact weight management) are problems (32). Thus, barriers to PCOS weight management over the lifespan include inadequate provider knowledge of PCOS, poor patient-provider communication and patients' lack of competency and self-efficacy for weight management.

Due to low-quality interactions with health providers during the PCOS diagnostic journey, peri-postmenopausal women with PCOS in our study sought information about the menopausal transition and PCOS as well as social support from online sources. The findings of others corroborate those in this study. In an analytical cross-sectional survey, researchers reported 98.2% of individuals with PCOS used Google to search PCOS symptoms and 18.8% sought support from others who understand PCOS (36). A cross-sectional online survey of peri-postmenopausal women without PCOS found that 77% of the respondents searched the internet for healthcare information (37). There are currently no studies that quantify the number of online searches for menopause with PCOS; however, there are many websites that offer information, indicating a demand for this topic.

### Sociocultural stigma

Cultural narratives influence societal norms, and societal norms shape personal identity and self-image through gender norms. Gender norms are societal expectations and rules about a woman's behavior (gender roles), presentation (appearance), and interactions (relationships) with others (38). Many cultures place primacy on fertility, motherhood, and conformity to prescribed ideas of femininity. For example, in India, newly married women are often given blessings by elders to produce many children. If the woman remains childless, she may lose the marital relationship and social status (39). In Western society, women who are overweight or have obesity face weight stigma and blame, as they defy the sociocultural ideal of slenderness. The discordance between gender norms and the perceived loss of a feminine identity by women with PCOS results in PCOS-stigma-related stress, compromised self-image, and feeling of isolation (40, 41).

Menopause does not remedy PCOS. While menopause ends menstrual irregularity and discomfort, other PCOS symptoms such as hirsutism and obesity continue and new symptoms such as alopecia may appear. In fact, androgen levels remain stable or increase during menopause, while estrogen levels decrease significantly (12). In addition to persistent and new PCOS symptoms, peri-postmenopausal women also experience physiological changes innate to aging, which compounds PCOS-related stigma with gendered ageism (42). Many of the participants expressed feelings of invisibility, which is consistent with the literature about older women. Social invisibility describes feelings of being devalued and becoming irrelevant as a woman and a person (43–45). In a mixed methods study involving peri-postmenopausal women from the United Kingdom, results revealed five themes: (1) feeling unseen, (2) being ignored, (3) being mis-seen, (4) being “grandmotherized”, and (5) being patronized (46). Thus, menopause (also synonymous with aging) compounds the detrimental effects of PCOS on self-image. These effects are as much about sociocultural stigmas as they are about physiological manifestations of PCOS and aging.

### Trauma experiences

Systemic and interpersonal trauma experiences among peri-postmenopausal women with PCOS can lead to medical distrust, nonadherence to medical treatment plans, and thus adverse health outcomes (24), as well as shame, emotional dysregulation, and unresolved grief (47). In recent years, the term “gaslighting” has been applied to the healthcare setting, referring to the denial, dismissal, and/or inadequate care of a patient by a healthcare provider or system (48). While this is the first study to apply the term to healthcare experiences among women with PCOS, previous research has examined the gaslighting phenomenon among women with chronic pain or diagnoses accompanied by stigmatization (e.g., HIV, fibromyalgia) (49–51), which is also associated with PCOS. Medical gaslighting is compounded among women with stigmatized chronic health conditions, which has been attributed to a patriarchal power structure in which women are stereotyped based on gender, race, and medical complaints (52). Institutional betrayal, another source of experiential trauma by peri-postmenopausal women with PCOS, is also a rapidly expanding area of research. A scoping review of 37 peer-reviewed articles over the last ten years revealed a direct link between perceived institutional betrayal and nonadherence with medical care (53). The findings of our study were consistent with the scoping review, as some participants avoided certain healthcare facilities or providers and/or disregarded the treatment plan.

The emotional toll of infertility and recurrent pregnancy losses created enduring trauma for the women in this study who described their experiences as sources of shame, unresolved grief, and depression. Shame was associated with perceived personal failure and the inability to fulfill expected gender roles. Participants attributed unresolved grief to healthcare dismissal at the time of miscarriage with many of the participants being discharged from an emergency department immediately at diagnosis without acknowledgement of the loss or resources to help cope with the loss. An integrative review of articles about miscarriage among women without PCOS revealed that women frequently report shame and grief for months to years (54). A review of studies found that 30%–50% of women with PCOS are at risk for a first miscarriage compared to 10%–15% of women without PCOS (54). The analysis of a nationwide, population-based database comparing pregnant women with PCOS to pregnant women without PCOS revealed that women with PCOS are more than three times more likely than women without PCOS to have recurrent pregnancy loss (55). The study also offered a longitudinal glance at how the compilation of unresolved trauma experiences intensifies depression. This may explain the finding from a cross-sectional survey that revealed peri-postmenopausal women had a better health-related quality of life than women with PCOS of reproductive age but moderate to high levels of depression (7).

### Self-advocacy

Participants discussed the importance of self-advocacy, especially for younger women starting their PCOS journey. In a scoping review of articles about patients' experiences with the PCOS diagnosis, the need for self-advocacy was identified as an essential skill for women with PCOS (56). Participants discovered a higher life meaning when helping younger women with PCOS develop assertiveness during encounters with healthcare providers. This finding was also consistent with findings of the previously referenced scoping review. Additionally, the finding aligns with a concept known as the “helper effect”. The “helper effect” is a principle that states people use the wisdom gained through living with a problem to help others with the same or similar problem, which increases life purpose and satisfaction (57). Paradoxically, participants in our study acknowledged a continuing struggle to self-advocate while seeking information and treatment during the menopausal transition. In the traditional patient-provider relationship, both parties view the provider as the expert and final authority in health-related concerns, which creates a power imbalance (58). The perceived power imbalance and the sociocultural norms for women (e.g., polite, accommodating) influence the confidence of older women to assert themselves during a healthcare encounter. Balancing the power differential requires that patients develop acceptance of diagnosis and autonomy in obtaining and processing health information while healthcare providers acknowledge and respect patients' experiential knowledge and include patients in the therapeutic process (59).

### Resilience vs. resignation

Consistent with the findings in our previous study involving the analysis of one open-ended question posed to peri-postmenopausal women with PCOS, women reported either active or passive coping (13), which aligned with the current study findings of resilience (active) or resignation (passive). Resilience is defined as the positive adaptation to significant stress and trauma, which is modulated by genetics, the environment, and individual characteristics and behaviors (26). Participants with resilience enact positive coping strategies (e.g., optimism, positive reframing, humor, physical activity). Women with PCOS who enact problem-focused coping vs. emotion-focused coping were found to exhibit more resilience (60). A systemic review and meta-analysis revealed that chronic illness can threaten resilience; however, individuals who actively self-manage are more likely to develop resilience over time (61). In a cross-sectional quantitative study involving women with PCOS aged 18–40 years, fertility-related stress and childlessness were negatively correlated with resilience, with a cultural background being a significant moderator (62). Findings from studies involving patients with other chronic conditions such as cancer and diabetes indicate that resilience can be developed or enhanced through training (63, 64).

Resignation is described in scientific literature as the end-stage response to long-term experiences of social threat, which can include perceived social exclusion and rejection, bullying, and discrimination (65). Resignation is, therefore, the passive acceptance of unfavorable conditions. Prolonged exposure to stress and trauma suppresses the ability to persevere and breeds low self-esteem, feelings of helplessness, and depression (66). In a cross-sectional study comparing women with PCOS with women without PCOS, women with PCOS who had depression and hirsutism were less likely to use active coping in stressful situations or seek emotional or instrumental social support (67). Findings from studies examining the influence of chronic illness revealed that perceived illness severity, especially when coupled with lack of social support, predicted resignation (27). One's adoption of sociocultural gender roles can also be an influential factor, which is most noticeable in women (68). Multidisciplinary programs are needed that help women with PCOS develop facilitators of resilience such as positive self-image, healthy lifestyle behaviors, quality social support, and self-management skills.

### Implications

#### Education and practice for healthcare professionals

The findings from our study have several implications for healthcare professionals in training and those in practice. The identified themes in this study highlight the need for a more proactive, trauma-informed, culturally tailored approach to women's health that provides education and support in managing PCOS over the lifespan. We advocate the use of the recently updated international guidelines for the treatment and management of PCOS, which also emphasize the need for improved patient-provider communication (2). Healthcare professionals require more training in using patient-centered communication techniques, such as respectful, active, and empathetic listening and proper acknowledgement of patient concerns. Patient-centered communication techniques can also mitigate the patient-provider power imbalance and help integrate women with PCOS as members of their own healthcare teams. To promote interactive health literacy among women with PCOS across the lifespan, AskPCOS is an app-based resource that provides checklists, prompting questions for healthcare providers, and health information about long-term health considerations (69).

Healthcare professionals also need continuous educational updates about PCOS, menopause, and menopause in the context of PCOS. Many healthcare professionals acknowledged the challenge of PCOS management and the need for better quality evidence and clinical guidelines and updated training. In a systematic review of healthcare providers' perspectives of PCOS management across the lifespan, eight articles were located that identified the weakness of clinical evidence and systemic barriers such as siloed care and lack of knowledge and training (70). Online resources, including those that are free of charge, are available to advance provider education. For example, The Ohio State University currently offers a free webinar for 1.00 credit of continuing education through February 17, 2026 (https://ccme.osu.edu/continuing-medical-education/webcasts/988/polycystic-ovary-syndrome/2/17/2023) and the Food and Drug Administration has a 1 h continuing education YouTube video posted in 2023 titled, *Polycystic Ovary Syndrome (PCOS) Revisited: Diagnosis, Management, and Future Needs*.

Throughout their lifespan, including during the menopausal transition, women with PCOS desire accurate and comprehensive medical information and appropriate resources for self-management and coping strategies to build resilience. These insights underscore the necessity for comprehensive mental health support and counseling services tailored to individuals experiencing infertility, miscarriages, and recurrent pregnancy loss. More tools are needed that enhance both interactive and functional health literacy among peri-postmenopausal women with PCOS. Our findings underscore the urgent need for healthcare systems to adopt more empathetic and patient-centered approaches to improve the care and support for women with PCOS. Many resources are available for individual healthcare providers as well as individuals who desire to improve their communication skills. For example, the Center for Health Care Strategies with support from the Robert Wood Johnson Foundation has a Trauma-Informed Care Resource Center that can support health systems to become a trauma-informed organization (https://www.traumainformedcare.chcs.org/what-is-trauma-informed-care/).

#### Research

Nearly all PCOS research involves women in their reproductive years. As a relatively unexplored area of women's health, more research is needed to address the needs of older women with PCOS. Longitudinal research is needed to improve the understanding of the PCOS trajectory throughout a woman's lifespan. Research is needed to develop and test programs that train healthcare providers to deliver patient-centered care and equip women with PCOS with the information and resources to properly manage PCOS and learn effective coping skills over the PCOS life course. Future research should explore interventions that promote effective patient-provider communication to build trust by eliminating medical gaslighting and institutional betrayal. In addition, as peri-postmenopausal women are searching the Internet, systems that assess the accuracy and quality of online healthcare information are needed.

### Limitations and strengths

Participants were recruited via ResearchMatch, which introduced selection bias, as most participants had at least a high school education, tend to be associated with health organizations or actively involved with community health events, and have Internet access. This may limit generalizability to the population of peri-postmenopausal women with PCOS. Nevertheless, as noted in the discussion, the findings concurred with the results of other qualitative studies involving women with PCOS, which provides some confidence in the generalizability. The sample was US-centric and lacked racial diversity as compared to the US Census Bureau's data. The sample size (*n* = 29) was a strength, as it provided rich, robust data and the first exploration of the physical, psychosocial, and healthcare perspectives of women with PCOS during and after the menopausal transition. Women self-reported PCOS and menopausal status. However, based on studies evaluating concordance between self-report and medical diagnoses, self-report has good concordance with electronic medical records and greater than 90% specificity for all medical diagnoses (71, 72). One strength of ResearchMatch is that it sends recruitment announcements to registrants based on the registrants' characteristics, including medical diagnoses. Thus, women who received the recruitment announcement had already identified themselves as having PCOS. Additionally, based on the women's responses to the interview questions, the researchers were confident that each participant had lived experiences with PCOS. A strength of the study was the use of COREQ that provided transparency and strengthened rigor and credibility of the results.

## Conclusion

The purpose of this interpretative qualitative study was to explore the physical, psychosocial, and healthcare perspectives of women with PCOS during and after the menopausal transition. As a chronic health condition, PCOS affects all stages of a woman's life from adolescence to menopause. Although a natural, inevitable life phase, little is known about the menopausal transition, especially in the context of PCOS. While the updated international guidelines for treating and managing PCOS include information for peri-postmenopausal women with PCOS, we highlighted further insight and need, such as trauma-informed care. Based on our findings, implications for education, practice and research were provided.

## Data Availability

The raw data supporting the conclusions of this article will be made available by the authors, without undue reservation.
