# Supplementary material for: “I feel like it gets worse as I get older”: perspectives of peri-postmenopausal women with PCOS
Source: Front Glob Womens Health. 2025 Aug 12;6:1588505. doi: 10.3389/fgwh.2025.1588505 (PMC12378712; doi:10.3389/fgwh.2025.1588505)
Supplement: Supplementary file 1 [file Table1.docx]

**Appendix A**

**Semi-Structured Interview Guide**

**Microsystem: self**

1. Describe your overall health, both physically and mentally.

How does PCOS influence your health?

How do you manage PCOS symptoms?

1. Describe how your perceptions of PCOS have changed over time, if at all.

What about PCOS, if anything, has stayed the same?

**Mesosystem: social supports and communities**

1. Tell me about your social support system.

What types of support have you found useful?

Who provided that support?

Probe: family, friends, or neighbors, organizations (e.g., church, clubs, or online groups)

What challenges have you had obtaining support?

Are there any types of support that you wish you had?

**Exosystem: social media, research funding, insurance**

1. Describe your sources of information on PCOS? On menopause?
2. If you could change anything about healthcare, what would it be and why?

Probe: Insurance, making appointments with specialists, interactions with providers

**Macrosystem: cultural norms**

1. As a woman, how have your roles (e.g., child, student, mother, employee) changed over time?
2. How do you view your future as a peri- or postmenopausal woman with PCOS?

**Chronosystem: historical context**

1. What does aging mean to you? Healthy aging?
2. Looking back, what would you like to tell your younger self?
3. What are your thoughts about recent political and cultural changes relating to women’s reproductive health?
4. Is there anything else I should have asked or that you would like me to know?

**OTHER**

1. May I email you about future PCOS studies for which you may be eligible?
